# Supplementary material for: Serine Protease HtrA2/Omi Deficiency Impairs Mitochondrial Homeostasis and Promotes Hepatic Fibrogenesis via Activation of Hepatic Stellate Cells
Source: Cells. 2019 Sep 20;8(10):1119. doi: 10.3390/cells8101119 (PMC6829542; doi:10.3390/cells8101119)
Supplement: Supplementary file 1 [file cells-08-01119-s001.pdf]

Supporting Information Text

Serine protease HtrA2/Omi deficiency impairs mitochondrial homeostasis and promotes hepatic fibrogenesis via activation of hepatic stellate cells

Short title: HtrA2/Omi deficiency promotes hepatic fibrogenesis

Wonhee Hur[1], Byung Yoon Kang[1], Sung Min Kim[1], Gil Won Lee[1], Jung-Hee Kim[1], Min-Kyung Nam[2], Hyangshuk Rhim[2] and Seung Kew Yoon[1*]

[1]The Catholic University Liver Research Center, College of Medicine, The Catholic University of Korea, Seoul, Republic of Korea

[2]Department of Biomedicine and Health Sciences, College of Medicine, The Catholic University of Korea, Seoul, Republic of Korea

[*]Correspondence to: Seung Kew Yoon, M.D.
Address: Department of Internal Medicine, Seoul St. Mary's Hospital
#222 Banpo-daero, Seocho-gu, Seoul, 137-701, Republic of Korea
Phone number: +82-2-2258-7534
FAX: +82-2-536-9559
E-mail: yoonsk@catholic.ac.kr

Gene Name	Sense primer	Antisense primer
mt-COX1	5'-CGG AGC CCC AGA TAT AGC ATT CCC ACG AAT-3'	5'-TCC TGT TCC TGC TCC-3'
mt-ND1	5'-TGC ACC TAC CCT ATC ACT CA-3'	5'-TCC TGA TCA TAG AAT GGA GT-3'
Ndufv1	5'-CTT CCC CAC TGG CCT CAA G-3'	5'-CCA AAA CCC AGT GAT CCA GC-3'
ATP5A	5'-AGG CCC TTT GCC AAG CTT-3'	5'-TTC TCC TTA GAT GCA GCA GAG TAC A-3'
COX5B	5'-GCT GCA TCT GTG AAG AGG ACA AC-3'	5'-CAG CTT GTA ATG GGT TCC ACA GT-3'
β-actin	5'-TTC CAG CCT TCC TT-3'	5'-ATC TCC TTC TGC ATC CTG TC-3'
mtDNA-79bp	5'-CAG CCG CTA TTA AAG GTT CG-3'	5'-CCT GGA TTA CTC CGG TCT GA-3'
mtDNA-230bp	5'-CAG CCG CTA TTA AAG GTT CG-3'	5'-GGG CTC TGC CAT CTT AAC AA-3'
18S	5'-AGT CCC TGC CCT TTG TAC ACA-3'	5'-GAT CCG AGG GCC TCA CTA AAC-3'

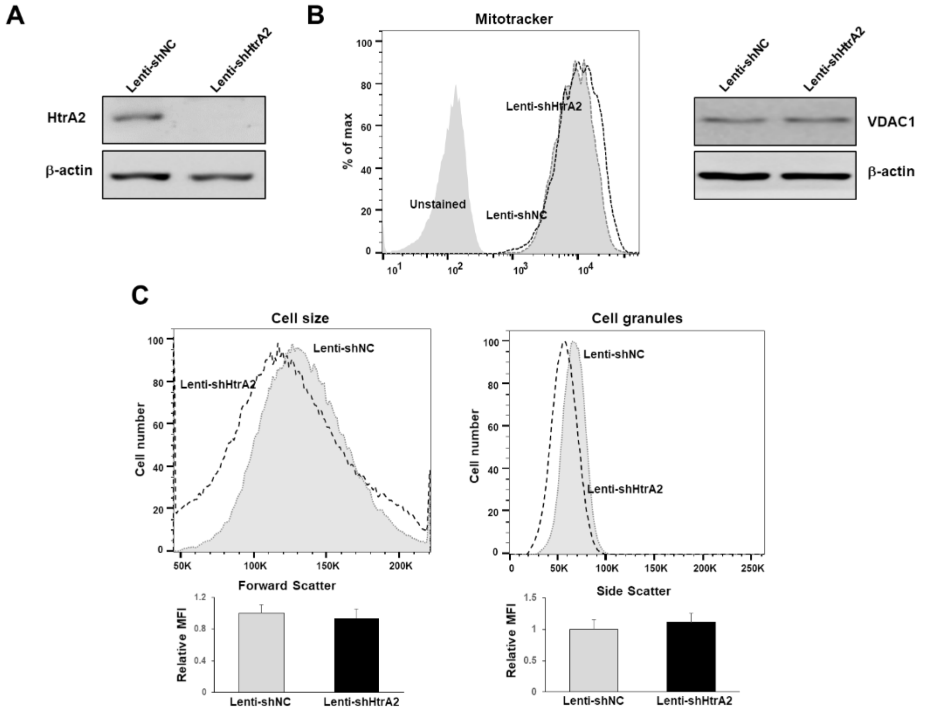

Figure S1. Lentivirus-mediated HtrA2/Omi depletion in hepatocyte lead to impaired mitochondrial function and metabolism. (A) HtrA2-shRNA encoding lentivirus were infected at MOI of 5 into FL83B cells. HtrA2/Omi protein levels were assayed using western blotting in lenti-shHtrA2 and lenti-shNC hepatocyte. (B) Mitochondrial morphology was measured by FACS-analysis using MitoTracker green staining. Western blotting for VDAC, mitochondrial mass proteins in lenti-shHtrA2 and lenti-shNC hepatocyte, with β-actin as a loading control. (C) Exemplary flow cytometry plot and histogram of showing populations of primary hepatocyte in light scatters; FSC and SSC. The fluorescence mean intensity of FSC and SSC was quantified.

Supplementary Fig. 2. Hur, *et al.*

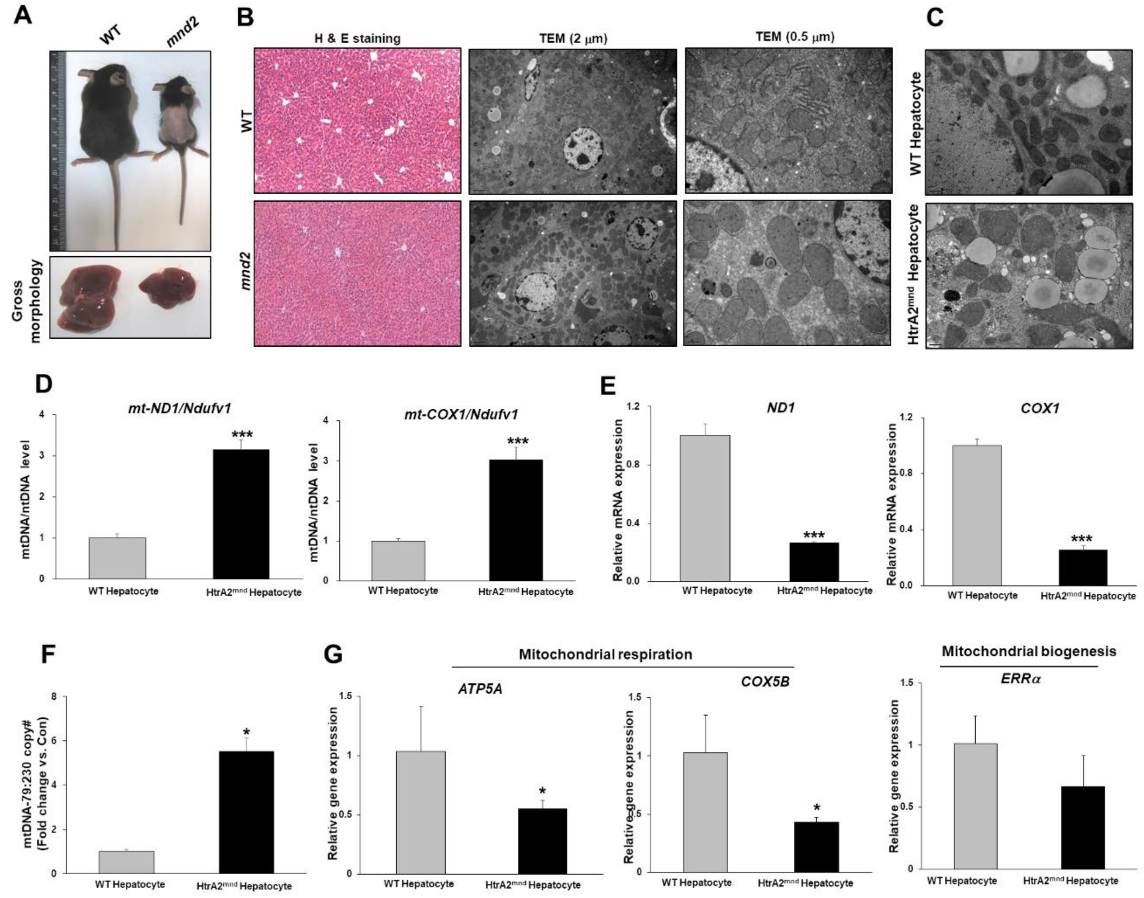

Figure S2. Loss of HtrA2/Omi in hepatocyte results in the accumulation of dysfunctional mitochondria and oxidative stress. (A) Changes of mitochondrial ultrastructure in liver tissue from WT or mnd2-mutant mice at postnatal day 40. Appearance of normal (WT, left panel), mnd2-mutant (right panel) mice and liver. (B) H&E staining (original magnification, X200) and TEM images of liver sections. (C) Representative TEM images of hepatocytes isolated from WT and mnd2-mutant mice. (D) qRT-PCR analysis of *mt-ND1* and *mt-COX1* DNA normalized to nuclear *Ndufv1* DNA in primary hepatocyte. (E & G) *ND1*, *COX1*, *ATP5A*, *COX5B* and *ERRα* mRNA expression in hepatocyte, as determined by qRT-PCR. (F) Damaged mtDNA levels of the 79 bp fragment (damaged) and 230 bp fragment (undamaged) in gDNA isolated from hepatocyte of mice were assessed by qPCR. Bars represent mean copy number ratios of mtDNA-79:230, normalized to 18S levels.

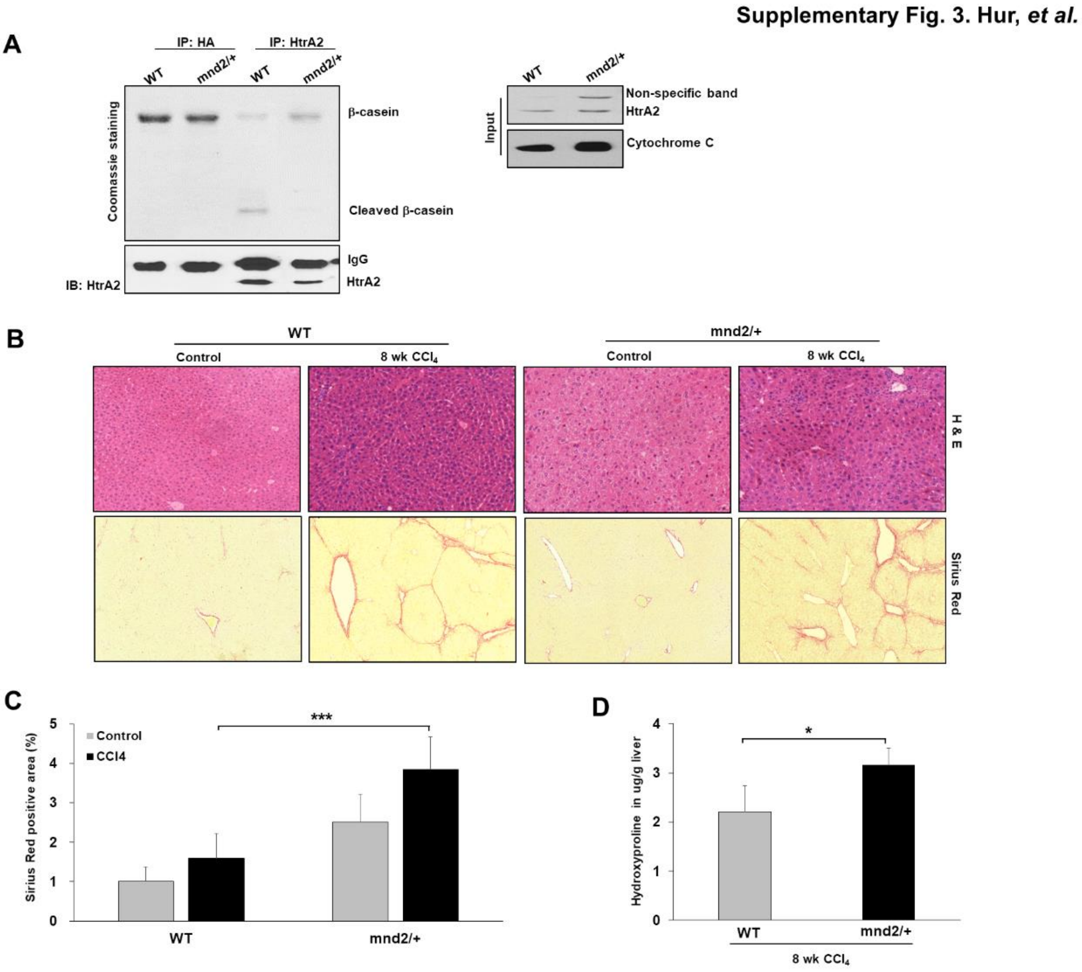

Figure S3. Loss of HtrA2/Omi mitochondrial protease activity promotes liver fibrosis. (A) Loss of serine protease activity of HtrA2/Omi in liver sections from WT and mnd2 heterozygous (mnd2/+) mice. Liver lysates were immunoprecipitated (IP) with HtrA2/Omi-specific polyclonal antibody. The IP complexes were incubated for the indicated times at 37 °C with β-casein as a substrate. The reaction samples were resolved by 15% SDS-PAGE, and the processing pattern of β-casein was visualized by staining with Coomassie Brilliant Blue dye (CBB). The level of the HtrA2 was analyzed by IB with HtrA2/Omi Ab. (B) Representative images of H&E and Sirius red staining of liver sections from WT and mnd2 heterozygous (mnd2/+) mice via repetitive CCl_4 injections (original magnification, X200). (C) Semi-quantitative analysis of Sirius red staining in the fibrotic livers. (D) The hydroxyproline content.

Supplementary Fig. 4. Hur, *et al.*

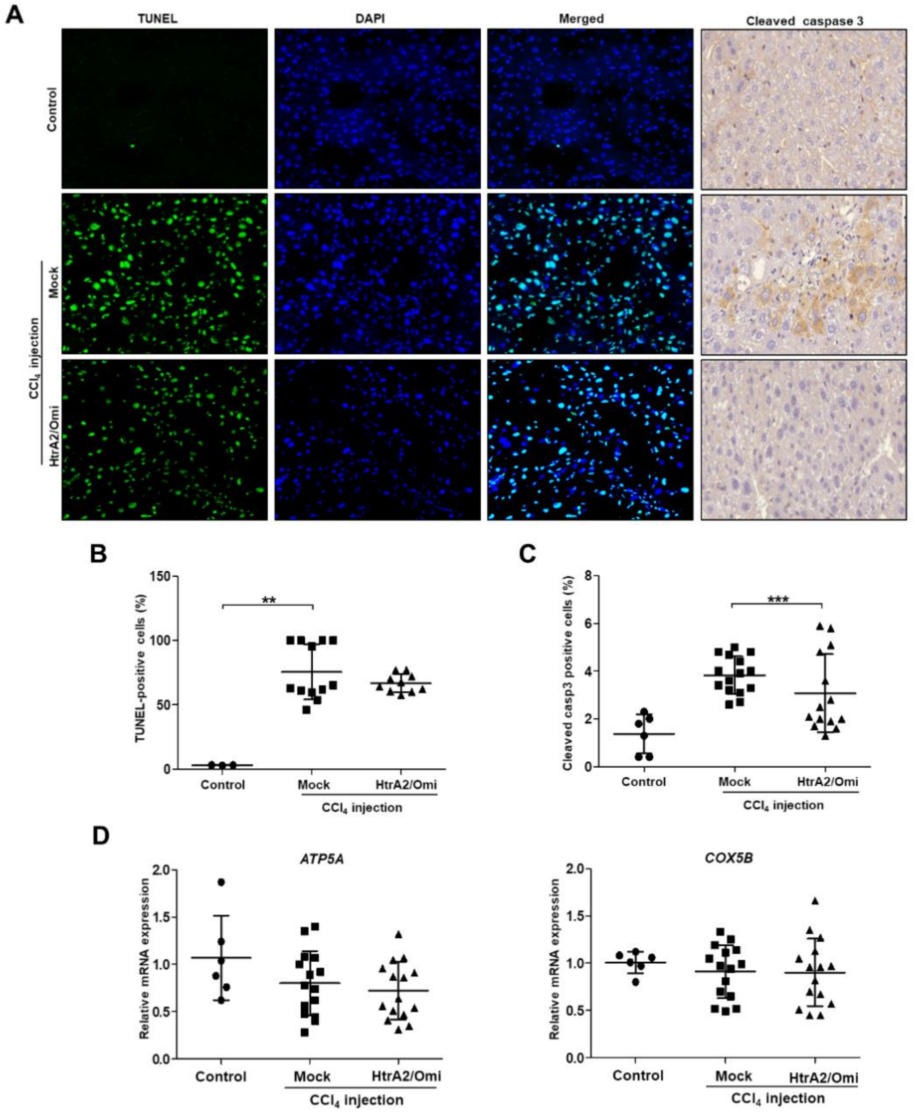

Figure S4. HtrA2/Omi expression in mouse model of CCl_4-induced liver fibrosis alleviates liver fibrosis by protecting hepatocytes damage. HtrA2/Omi expression in CCl_4-treated mice following the hydrodynamic tail vein injection of HtrA2/Omi-encoding plasmid DNA (n=7) compared with the mock control group (n=5) over 8 weeks. Hepatic fibrosis was induced by injection of CCl_4 two times per week for 8 weeks. (A&B) HtrA2/Omi expression alleviates CCl_4-induced hepatocyte death. Representative pictures of TUNEL and activated caspase 3 staining for apoptosis detection. Nuclei were stained with DAPI (blue). (C) The immunohistochemistry staining for cleaved caspase-3 was quantified by Image-Pro Plus 6 software. (D) *ATP5A* and *COX5B* mRNA expression in liver tissue, as determined by qRT-PCR.
